# Artificial intelligence in primary aldosteronism: current achievements and future challenges

**DOI:** 10.3389/fmolb.2025.1660588

**Published:** 2025-08-21

**Authors:** Yisi Xu, Benjin Liu, Xuqi Huang, Xudong Guo, Ning Suo, Shaobo Jiang, Hanbo Wang

**Affiliations:** Department of Urology, Shandong Provincial Hospital Affiliated to Shandong First Medical University, Jinan, China

**Keywords:** primary aldosteronism, artificial intelligence, machine learning, predictive model, diagnosis

## Abstract

Recent advances in artificial intelligence (AI) are reshaping the diagnostic and therapeutic of primary aldosteronism (PA). For screening, machine learning models integrate multidimensional data to improve the efficiency of PA detection, facilitating large-scale population screening. For diagnosis, AI-driven algorithms have further enhanced the specificity of PA identification. In subtype classification, AI algorithms achieve high predictive accuracy in differentiating PA subtypes through comprehensive analysis of clinical, imaging, and biochemical data, while simultaneously reducing reliance on invasive diagnostic procedures. Regarding treatment decision-making and outcome, predictive models guide personalized therapy by assessing treatment responses and surgical results. These models also contribute to discovering potential drugs by analyzing molecular targets computationally. Although scientists have achieved notable progress, there remain substantial challenges in clinical implementation, including limited sample size, insufficient model interpretability, and a lack of real-world validation. To translate technical advances into clinical practice, the field requires more reliable AI models with clear decision-making processes and rigorous multicenter validation studies. Future research should focus on clinical practice by developing integrated diagnostic-treatment pathways, while leveraging AI’s strengths and overcoming its current limitations in generalizability and clinical acceptance.​

## 1 Introduction

Primary aldosteronism (PA)​, a critical form of endocrine hypertension, was first systematically described by Conn in 1955 (Conn, 1955). Diagnostic improvements have established PA as the predominant endocrine cause of secondary hypertension (Stowasser and Gordon, 2016). PA typically results from excessive and unregulated aldosterone secretion, with pathological subtypes mainly including aldosterone-producing adenoma (APA), idiopathic hyperaldosteronism (IHA), as well as rare cases of unilateral adrenal hyperplasia, familial hyperaldosteronism, and adrenal carcinoma. Compared to essential hypertension, PA patients exhibit more significant target organ damage and show higher risks of cardiovascular disease and metabolic abnormalities when matched for blood pressure levels (Reincke et al., 2021; Dogra et al., 2023; Mullen et al., 2024).

Epidemiological studies report the prevalence of PA is 4%–11.2% in hypertensive populations (Rossi et al., 2006; Xu et al., 2020; Funder, 2022; Scholl, 2022; Ekman et al., 2024), and can up to 20% in those with treatment-resistant hypertension (Byrd et al., 2018; Reincke et al., 2021). However, growing research indicates that the incidence may be substantially underestimated, as only a few at-risk populations were screened for this disease (Gouli et al., 2011; Brown et al., 2020; Funes Hernandez and Bhalla, 2023). The diagnosis of PA follows the following protocol: screening, confirmatory testing, and crucial differentiation between unilateral and bilateral subtypes (Reincke et al., 2021). However, this multi-stage process poses challenges to the allocation of medical resources and clinical compliance. Making matters worse, PA’s nonspecific clinical manifestations and the limited emphasis on PA screening in current hypertension guidelines lead to significant knowledge gaps among clinicians (Funder and Carey, 2022; Mullen et al., 2024), which undoubtedly further increases the difficulty of clinical identification. Together, these challenges create a disparity between the detection rate and the actual incidence of PA, highlighting the necessity for improved clinical decision-support systems.

In recent years, artificial intelligence (AI) is transforming medical research toward precision medicine through its advanced pattern recognition and multidimensional data analysis capabilities, providing a new solution to current medical challenges. The key technology driving this transformation is machine learning (ML), which includes deep learning (DL), natural language processing (NLP), and computer vision (Padmanabhan et al., 2021; Dossabhoy et al., 2023). In the field of PA, AI models exhibit remarkable potential to overcome limitations of traditional approaches in ​essential aspects​: screening and diagnosis optimization, disease subtyping and localization, as well as treatment decision-making and management.

In this review, we focus on the innovative applications of AI in the diagnosis and treatment of PA, highlighting its comparative advantages over conventional approaches. The findings demonstrate that AI technologies significantly improve diagnostic efficiency and subtype classification accuracy through multimodal data integration, offering a novel pathway for precision medicine in PA management. Meanwhile, we also identify current research limitations and provides direction for future investigations.

## 2 Screening and diagnostic optimization

Table 1 presents representative studies on screening and diagnostic optimization, organized sequentially as discussed in this section, with details on algorithm, predictors, and AUC values.

**TABLE 1:** Screening and diagnostic optimization.

References	Algorithm	Predictors	AUC
Lin et al. (2022)	—	age, sex, hypokalemia, serum sodium, serum sodium-to-potassium ratio, anion gap, and alkaline urine	0.839 (training)
			0.814 (internal validation)
			0.839 (external validation)
Buffolo et al. (2021)	RFr	sex, systolic blood pressure, antihypertensive medication daily dosage, BMI, lowest potassium level, and target organ damage	0.834 (PA)
			0.905 (UPA)
Constantinescu et al. (2024)	—	aldosterone, 18-oxocortisol, and 18-hydroxycortisol	0.848
Eisenhofer et al. (2020)	RF and SVM	aldosterone and 18-oxocortisoletc.	0.926
Wilkes et al. (2018)	RF, WSRF, and XGBT	—	0.955 (WSRF)
Prete et al. (2024)	RF and GMLVQ	3α,5β-tetrahydroaldosterone, tetrahydro-11-deoxycortisol, and 18-hydroxy-tetrahydro-11-dehydrocorticosterone	0.950 (RF)
			0.960 (GMLVQ)
Burrello et al. (2021)	LR, RF, SVM and LDA	male sex, antihypertensive medication, plasma renin activity, aldosterone levels, potassium levels, and the presence of organ damage	0.879 (training)
			0.877 (internal validation)
Kaneko et al. (2019)	LightGBM	aldosterone levels, serum potassium levels, and adrenal nodule diameter	0.850 (combined)
			0.813 (SIT)
			0.786 (CCT)
Wu et al. (2023)	—	—	0.935

### 2.1 Aldosterone-to-renin ratio

The measurement of plasma aldosterone concentration (PAC) and renin activity to calculate the aldosterone-to-renin ratio (ARR) remains the most recommended screening method for PA (Funder et al., 2016; Mulatero et al., 2020; Reincke et al., 2021). However, this method requires complex biochemical assays and specialized equipment, limiting its feasibility for large-scale screening and contributing to high rates of underdiagnosis. To address this issue, Lin et al. developed an online PA prediction model using a supervised learning algorithm, which incorporated seven key predictors: age, sex, hypokalemia, serum sodium, serum sodium-to-potassium ratio, anion gap, and alkaline urine (Lin et al., 2022). The model showed strong diagnostic performance across training, internal validation, and external validation cohorts with area under the receiver operating characteristic curve (AUC) values of 0.839, 0.814, and 0.839. Remarkably, this model utilizes routine clinical parameters, eliminating the need for complex hormonal testing. Through the DxAI platform, it provides clinicians with an interactive web-based tool that transforms complex ML algorithms into a physician-friendly interface. This approach provides primary care facilities with a reliable and convenient alternative for PA screening, which significantly improves screening feasibility. Similarly, Buffolo et al. constructed a random forest regression (RFr) model with easily obtainable clinical parameters, including sex, systolic blood pressure, antihypertensive medication daily dosage, body mass index (BMI), lowest potassium level, and target organ damage (Buffolo et al., 2021). Their RFr model demonstrated AUC values of 0.834 for PA detection and 0.905 for unilateral PA (UPA) identification, potentially preventing unnecessary screening in up to 32.7% of hypertensive patients. However, both studies used retrospective designs, lacking prospective clinical validation and the economic evaluation compared to ARR testing. Besides, Lin et al. excluded the patients with comorbidities (e.g., diabetes, cancer), which may limit the model’s generalizability to real-world heterogeneous populations.

### 2.2 Plasma steroids

Current guidelines recommend discontinuing interfering medications before ARR measurement to ensure diagnostic accuracy (Funder et al., 2016). Specifically, antihypertensive drugs such as angiotensin-converting enzyme inhibitors (ACEIs), angiotensin receptor blockers (ARBs), diuretics, and mineralocorticoid receptor antagonists (MRAs) can interfere with renin and aldosterone levels by affecting the renin-angiotensin-aldosterone system, which may lead to false-negative or false-positive ARR results (Mulatero et al., 2002; Seifarth et al., 2002; Eide et al., 2004; Lamarre-Cliche et al., 2005; Ahmed et al., 2010; Huang et al., 2013; Volpe et al., 2013; Guo et al., 2020). However, withdrawing these medicines may induce dangerous blood pressure fluctuations and the medication adjustment is difficult in clinical practice, especially for patients with comorbidities (Funder et al., 2016; Dogra et al., 2023; Funes Hernandez and Bhalla, 2023). Constantinescu et al. developed a steroid probability score using advanced plasma steroidomics technology to overcome this constraint (Constantinescu et al., 2024). They applied ML algorithms to analyze comprehensive steroid profiles measured by mass spectrometry, including aldosterone, 18-oxocortisol, and 18-hydroxycortisol. Receiver operating characteristic analysis revealed superior diagnostic performance (AUC = 0.848) compared to ARR (AUC = 0.765) in patients receiving concurrent antihypertensive therapy (including ACEIs, ARBs, β-blockers, dihydropyridin calcium channel blockers, and diuretics), indicating its better pharmacological interference resistance. Multivariable regression confirmed minimal impact from most antihypertensives (P > 0.05) except MRAs and diuretics, offering a reliable screening alternative when medication adjustment is impractical. Notably, this score builds upon Eisenhofer’s earlier framework, which first proposed the integration of steroid profiling with ML (Eisenhofer et al., 2020; Constantinescu et al., 2024). They employed random forest (RF) and support vector machine (SVM) algorithms to analyze plasma steroid profiles (Eisenhofer et al., 2020). Their research demonstrated that RF models incorporating seven key steroids achieved significantly better PA detection (AUC = 0.926) than ARR (AUC = 0.890; ΔAUC = 0.036, P = 0.003) (Eisenhofer et al., 2020). Constantinescu’s study provided clinical validation for the steroid profiling-machine learning framework developed by Eisenhofer’s team, driving PA diagnosis toward efficient and minimally disruptive detection approaches. However, the technology’s reliance on Liquid Chromatography-Tandem Mass Spectrometry limits its applicability in resource-limited primary care settings.

### 2.3 Urinary steroid

Emerging evidence suggests urinary steroid metabolite analysis may offer clinical advantages over plasma-based approaches for endocrine evaluation (Araujo-Castro et al., 2021; Prete et al., 2024): (1) it enables comprehensive evaluation of 24-h adrenal steroid secretion, better reflecting total hormone production without transient serum level fluctuations; (2) this approach exhibits enhanced analytical stability, remaining unaffected by postural variations (seated vs supine) that alter serum steroid profiles; (3) as a ​noninvasive procedure, it eliminates the need for venipuncture. Wilkes et al. employed 3 ML approaches—RF, weighted-subspace random forest (WSRF), and extreme gradient boosted tree (XGBT)—to automatically analyze urinary steroid profiles (Wilkes et al., 2018). The WSRF algorithm showed superior performance in distinguishing normal urinary steroid profiles​ (all metabolite concentrations/ratios within age/sex-matched reference intervals) and abnormal profiles​ (≥1 metabolite/key ratio beyond reference ranges, indicating adrenal pathology), achieving an AUC of 0.955 with both sensitivity and specificity exceeding 90%. While the RF model is the most reliable in discriminating diverse adrenal-related abnormal biochemical patterns, achieving a balanced accuracy of 0.873. Their study provides a methodological reference for developing multiparameter diagnostic models in adrenal disorders like PA. Nevertheless, the normal/abnormal classification of all training data was based on clinician’s subjective judgment rather than objective criteria such as histology or genetic testing. The model only predicted abnormal biochemical patterns and cannot directly correspond to specific disease diagnoses, resulting in a disconnect from clinical diagnostics. A study by Prete’s team focused on the precision diagnosis of PA (Prete et al., 2024). They​​ applied ​generalized matrix learning vector quantization (GMLVQ)​​ and ​RF algorithms to analyze urine steroid metabolomics data, identifying key biomarkers for diagnosing ​PA: 3α,5β-tetrahydroaldosterone, tetrahydro-11-deoxycortisol, and 18-hydroxy-tetrahydro-11-dehydrocorticosterone (Prete et al., 2024). The results demonstrated these three metabolites could accurately distinguish PA patients from healthy controls, with AUC values of 0.960 (GMLVQ) and 0.950 (RF). Remarkably, the GMLVQ model identified ​18-oxo-tetrahydrocortisol as a specific biomarker for diagnosing *KCNJ5*-mutated APAs (AUC = ​ 0.85)​. Since these patients exhibit superior postoperative outcomes, the model provides them with direct guidance for prioritizing adrenalectomy. However, this model exhibited suboptimal performance in subtype classification (AUC = 0.65) and lacked prospective validation.

### 2.4 Confirmatory test

As a screening tool, ARR demonstrates adequate sensitivity but suffers from limited specificity, resulting in false positive rates of 30%–50%, while confirmatory tests effectively distinguish PA from false positive cases (Morera and Reznik, 2019). International guidelines emphasize confirmatory testing as an essential part of PA diagnosis, except in cases with specific hyperaldosteronism features (e.g., plasma aldosterone >20 ng/dL with spontaneous hypokalemia) (Funder et al., 2016). Currently, the main confirmatory tests include the saline infusion test (SIT), captopril challenge test (CCT), oral sodium loading test, and fludrocortisone suppression test, among which SIT and CCT are most widely adopted internationally (Reincke et al., 2021). However, the complexity of these tests, combined with inconsistencies between guidelines in recommendations and diagnostic thresholds, significantly hinders PA diagnosis. Burrello et al. developed a ML-based Primary Aldosteronism Confirmatory Testing (PACT) scoring system, using logistic regression (LR) and 3 ML algorithms—RF, SVM, and linear discriminant analysis (LDA)—to identify six key predictors: male sex, antihypertensive medication, plasma renin activity, aldosterone levels, potassium levels, and the presence of organ damage (Burrello et al., 2021). Their 16-point scoring system classifies patients into low (<5 points), intermediate (5–12 points), and high-risk (≥13 points) categories: low-risk individuals can reliably exclude PA without confirmatory testing, high-risk cases proceed directly to subtype differentiation, while only intermediate-risk patients require conventional confirmatory tests. The AUC of this scoring model was 0.879 in the training set and 0.877 in the internal validation set, potentially eliminating confirmatory testing for 22.8% of cases, with 95%–100% accuracy for unilateral PA identification. Furthermore, the PACT score parameters are typically accessible in primary care settings, demonstrating good applicability in community healthcare. However, this study omitted evaluation of ethical concerns when high-risk patients bypassed confirmatory testing for direct surgical intervention.

Additionally, the clinical value of combining multiple confirmatory tests remains controversial. While some guidelines advocate dual-test protocols for enhanced specificity (Nishikawa et al., 2011), multicenter studies suggest single test already provides sufficient predictive ability (Rossi et al., 2007; Kaneko et al., 2019; Wada et al., 2021). Kaneko et al. employed a variant of gradient boosting—light gradient boosting machine (LightGBM) model to analyze biochemical indicators from SIT and CCT alongside clinical features (aldosterone levels, serum potassium levels, and adrenal nodule diameter), and found that there was no statistical difference in predictive performance between combined testing (AUC = 0.850) and individual test (SIT: AUC = 0.813; CCT: AUC = 0.786, P > 0.05) (Kaneko et al., 2023). Although this Japanese retrospective study provides valuable evidence for simplifying the PA classification process, its generalizability requires validation in diverse populations with standardized testing protocols.

### 2.5 Electronic health record

Currently, the clinical identification of PA faces dual challenges. On one hand, in primary hospitals, traditional diagnostic procedures often lead to insufficient screening, resulting in missed diagnoses, or excessive reliance on comprehensive screening, causing unnecessary medical resource consumption. On the other hand, existing ML approaches predominantly rely on structured numerical parameters, failing to effectively integrate unstructured textual data from electronic health record (EHR) (e.g., chief complaints, medical history) with laboratory results. Moreover, most models lack clinically logical and staged decision-making frameworks, which limit their practicality and transparency. Wu et al. addressed these gaps by utilizing NLP techniques to convert numerical indicators (e.g., renin activity, aldosterone levels) into medical descriptive text, subsequently integrating them with unstructured clinical narratives (Wu et al., 2023). They innovatively introduced a label embedding-attentive framework to enhance disease-specific feature extraction. This framework encodes disease diagnostic criteria from medical guidelines into label embeddings, then performs interactive attention computation with semantic representations of patient EHR texts to enhance the model’s ability to capture pathognomonic clinical features. Their model achieved an accuracy of 0.963 and an AUC of 0.935 for PA diagnostic, representing the first successful multimodal EHR-based intelligent PA discrimination system. While demonstrating promising accuracy, the model’s reliance on data from a single healthcare system needs multi-center validation. Its dependency on comprehensive laboratory results limits real-world clinical utility.

## 3 Subtyping and localization techniques

Table 2 lists key studies on subtyping and localization techniques in order of discussion, summarizing their algorithms, predictors, and AUC values.

**TABLE 2:** Subtyping and localization techniques.

References	Algorithm	Predictors	AUC
Mansour et al. (2023)	RF	—	0.56 (radiomics-only)
			0.67 (integrating multiple parameters)
Chen et al. (2024)	RF, FNN, NNet and KNN	—	0.778 (Stage I)
			0.831 (Stage II)
Shi et al. (2022)	RF	age, blood pressure, ARR, CCT, and post-SIT biochemical markersetc.	0.938 (internal testing)
			0.887 (external validation)
Kaneko et al. (2021)	RF, LR, SVM, and GBDT	serum potassium, sodium, and aldosterone concentration	0.950 (test cohort)
			0.826 (external validation)
Burrello et al. (2020a)	RF and LDA	aldosterone at screening and post-confirmatory, lowest potassium value, presence/absence of nodules at CT scanning, the diameter of largest nodule, and descriptive CT findings	—
Burrello et al. (2020b)	RF and LDA	aldosterone at screening and post-confirmatory, lowest potassium value, ipsilateral and contralateral imaging findings at CT scanning, and AVS contralateral ratios	—
Vékony et al. (2024)	NNet	hsa-miR-146a-5p+, hsa-miR-24-3p+, hsa-miR-130b-3p+, hsa-miR-99b-5p+, hsa-miR-151a-3p+, hsa-miR-199a-3p	0.87 (validation cohort)

### 3.1 Computed tomography

As a crucial imaging technique, computed tomography (CT) primarily used for adrenal morphological evaluation and subtype classification in the diagnostic process of PA. However, conventional CT has limitations in distinguishing PA subtypes and localizing functional adrenal tumors. It demonstrates insufficient sensitivity for micronodules (<1 cm) and difficulty in differentiates nonfunctional adrenal nodules from functional lesions (Young, 2019), which may lead to misdiagnosis or delayed treatment. Recent advances in ML-based radiomics models have shown significant promise for PA subtyping. Mansour et al. developed a predictive model combining clinical parameters and CT radiomic features to enhance subtype discrimination (Mansour et al., 2023). By using maximum-relevance minimum-redundancy (MRMR) algorithm, they selected 25 optimal radiomic features to establish a baseline RF model. The radiomics-only model achieved an AUC of 0.56, while incorporating baseline serum potassium levels and SIT results enhanced overall performance to 0.67 (Mansour et al., 2023). Although this study confirmed that integrating some clinical parameters can boost radiomic model performance, the single-stage RF framework demonstrates only moderate performance in multiclass classification tasks, with remaining limitations in lateralization determination for unilateral lesions. In later research, Chen et al. proposed an innovative two-stage ML model (Chen et al., 2024). They combined triphasic abdominal CT radiomics (107 morphological, density, and texture features) with 16 biochemical markers to construct a hierarchical classification framework. In Stage I, three models were compared for unilateral/bilateral discrimination: (1) a feedforward neural network (FNN) model using radiomic features, (2) a RF model using clinicobiochemical characteristics, and (3) an integrated model integrating both feature types. The integrated model showing optimal performance, achieving 80.6% accuracy (AUC = 0.778). Stage II utilized pure radiomics via a neural network (NNet) for laterality determination (accuracy = 88%, AUC = 0.831). The final integrated model achieved an accuracy of 77.5% and F1 score of 70.5%, significantly outperforming conventional CT interpretation by radiologists’ evaluation (Accuracy = 46.2%, F1 score = 41.2%, P < 0.05). In their study, only 61.4% of patients completed all three-phase CT scans, so they introduced K-nearest Neighbor (KNN) imputation to handle missing multiphase CT data. While KNN imputation offers value for incomplete medical imaging datasets, its potential bias introduction remains concerning. Regrettably, Chen et al. failed to quantitatively evaluate the imputation effects and the validation cohort is limited (n = 32), potentially compromising model reliability.

### 3.2 Adrenal venous sampling

Adrenal venous sampling (AVS) is the gold standard for subtyping PA, with its core value lying in distinguishing unilateral APA from bilateral IHA. This technique effectively compensates for the limitations of conventional CT, thereby guiding therapeutic decisions between adrenalectomy and lifelong medication (Shi et al., 2022). However, AVS implementation faces multiple challenges, including technical complexity, high costs, variable success rates due to adrenal vein anatomical variations, radiation exposure from invasive procedures, and dependence on experienced physicians (Young and Stanson, 2009; Rossi et al., 2014; Reincke et al., 2021; Zuo et al., 2023). These constraints have driven the researchers to explore non-invasive alternatives for PA subtyping. Shi et al. developed a RF model incorporating 10 non-invasive clinical parameters such as age, blood pressure, ARR, CCT, and post-SIT biochemical markers to discriminate UPA and bilateral PA (BPA) (Shi et al., 2022). The model achieved 90.0% accuracy (AUC = 0.938) in internal testing and 81.4% accuracy (AUC = 0.887) in external validation, potentially reducing AVS requirements by 51.2%. The model by Shi et al. achieves strong performance but requires specialized infrastructure, whereas Kaneko et al.'s streamlined approach facilitates ML integration at the primary care settings (Kaneko et al., 2021; Shi et al., 2022). They applied RF, LR, SVM, and gradient boosting decision trees (GBDT) to analyze 229 AVS-confirmed PA patients (Kaneko et al., 2021). The RF model demonstrated optimal performance for unilateral subtype prediction (accuracy = 95.7%, AUC = 0.990). Using Gini importance, they selected serum potassium, aldosterone, and the novel independent predictor sodium to construct a simplified model. The new model achieved 89.1% accuracy (AUC = 0.950) in testing and 85.1% (AUC = 0.826) in external validation. This innovative blood-based tool providing primary hospitals with an effective and low-cost strategy to identify high-risk patients in need of AVS.

While the studies mentioned above validated ML models using routine clinical and biochemical parameters for PA subtyping, Burrello et al. further emphasized the significance of imaging features, developing a clinically applicable scoring system beyond algorithmic models (Burrello et al., 2020a; Burrello et al., 2020b). They selected six key variables via multivariate regression analysis (aldosterone at screening and post-confirmatory, lowest potassium value, presence/absence of nodules at CT scanning, the diameter of largest nodule, and CT scan findings) to construct LDA and RF models for PA lateralization (Burrello et al., 2020b). The RF model showed higher classification efficacy (93.0% training, 87.0% validation accuracy). Then the ML outputs were converted to a 20-point clinical scale: scores ≤8 strongly suggested BPA (sensitivity = 97.8%), scores >16 indicated lateralized PA (specificity = 98.2%), and intermediate scores (8.5–16) required confirmatory AVS. The model achieved 89.3% accuracy in the training cohort and reduced unnecessary invasive procedures by 43.7%, with this reduction rate reaching 66.1% in the external validation cohort. This scoring system has undergone cross-institutional validation, offering a practical solution for regions with limited AVS resources. In subsequent work, they address unilateral AVS success with contralateral suppression, using LDA and RF model screened out vital variables: aldosterone at screening and post-confirmatory, lowest potassium value, ipsilateral and contralateral imaging findings at CT scanning (normal imaging: absence of nodules or with thickening <4 mm; abnormal imaging: presence of a mass ≥8 mm or thickening ≥4 mm), and AVS contralateral ratios (Burrello et al., 2020a). External validation achieved 84.6% (LDA) and 82.9% (RF) accuracy to classify BPA and UPA. They also constructed a 19-point score system based on these two predictive models that decreased AVS repetition rates by 77.2%, which is particularly valuable for institutions with limited AVS experience. The two studies demonstrate methodological continuity, combining ML algorithms with clinical scoring systems and proposing concrete implementation pathways. While overlapping patient cohorts help maintain consistency, they may introduce selection bias. Clinically, both models’ reliance on imaging findings presents a double-edged sword: while CT features are key predictors, their dependence on radiologist interpretation risks inter-observer variability across institutions. Additionally, like many studies, both studies utilized retrospective data, necessitating prospective multicenter research to improve the generalization ability of the models.

Furthermore, the combination of ML with molecular biomarkers offers a novel approach to solve clinical challenges in PA subtyping. Vékony et al. developed a non-invasive method to discriminate between UPA and bilateral adrenal hyperplasia by combining miRNA profiling with ML algorithms (Vékony et al., 2024). Their study analyzed microRNA sequencing data from adrenal venous samples of 18 patients, identifying six key microRNAs through a single-hidden-layer NNet, which were subsequently validated using a DL model. The model achieved perfect classification accuracy (AUC = 1.0) in 30 paired AVS-peripheral blood samples and maintained robust performance (AUC = 0.87) in an independent validation cohort of 108 cases. Remarkably, it successfully classified pathological subtypes in six cases with AVS lateralization index falling within the borderline range, demonstrating high diagnostic precision for PA subtyping while reducing reliance on AVS.

## 4 Therapeutic decision-making and prognostic evaluation

Table 3 organizes pivotal studies on therapeutic decision-making and prognostic evaluation, while documenting their respective algorithms, predictors, and AUC values.

**TABLE 3:** Therapeutic decision-making and prognostic evaluation.

References	Algorithm	Predictors	AUC
Kaneko et al. (2022)	GBDT and SHAP	hypertension duration <7 years, drug dosage <3 DDD, PAC ≥37 ng/dL, and BMI ≤22 kg/m^2^	0.884 (internal testing)
			0.867 (external validation)
Wang et al. (2021)	XGBoost	elevated 60-min insulin levels during preoperative OGTT, increased post-dexamethasone suppression corticosteroid levels	—
Yu et al. (2023)	LASSO, SVM-RFE and RF	SST, RAB3C, PPY, CYP3A4, and CDH10	0.755 (validation cohort)
Sun et al. (2025)	LASSO and RF	ATP2A3	0.879

### 4.1 Postoperative prediction

UPA is a form of endocrine hypertension that can be cured by unilateral adrenalectomy. However, the Primary Aldosteronism Surgical Outcome (PASO) study revealed that while most patients achieve biochemical success by correcting aldosterone oversecretion post-adrenalectomy, only 37% attain complete clinical success—defined as normalized blood pressure without antihypertensive medications (Williams et al., 2017). This highlights the need for personalized treatment strategies based on individual patient characteristics. Traditional approaches relying on generalized linear models, which often fail to capture complex nonlinear relationships among clinical predictors, and existing prediction models exhibit significant heterogeneity. To overcome these constraints, Kaneko et al. using GBDT to predict complete clinical success after adrenalectomy in UPA patients (Kaneko et al., 2022). Their study analyzed 107 biochemically successful cases, identifying six preoperative predictors: hypertension duration, defined daily dose (DDD), PAC, sex, BMI, and age. Using Shapley additive explanations (SHAP) algorithm, they quantified the contribution of each feature to the model and determined nonlinear predictive thresholds—most notably hypertension duration <7 years, drug dosage <3 DDD, PAC ≥37 ng/dL, and BMI ≤22 kg/m^2^. The model demonstrated robust performance in internal testing (77.3% accuracy, AUC = 0.884) and external validation (80.4% accuracy, AUC = 0.867), providing a practical tool for individualized surgical benefit assessment. Nevertheless, the model’s generalizability is currently constrained by the homogeneous Japanese validation cohort and the model assessment potentially lacks statistical power with only 22 test samples in internal testing. Future multicenter studies incorporating diverse ethnic populations, along with imaging or molecular biomarkers, are needed to enhance predictive accuracy.

Although most patients with UPA maintain normal adrenal function after adrenalectomy, nearly 27% of them develop adrenal insufficiency, typically manifested as impaired postoperative cortisol secretion (Heinrich et al., 2019). To investigate the risk of this condition in unilateral APA patients, Wang et al. constructed classification and regression models using ML algorithms including extreme gradient boosting (XGBoost) and model-based boosting methods based on 78 preoperative variables (Wang et al., 2021). The study revealed that elevated 60-min insulin levels during preoperative oral glucose tolerance test (OGTT) positively correlated with post-stimulation cortisol, while increased post-dexamethasone suppression corticosteroid levels showed negative correlation, suggesting glucocorticoid co-secretion as a potential risk factor for postoperative adrenal insufficiency (Wang et al., 2021). However, limitations including small and imbalanced samples, high-dimensional variables, and lack of independent validation cohorts resulted in significantly compromised model generalizability (cross validation showed *R* ^2^ decline from 0.80 in training to 0.18), indicating unreliable clinical predictability with current data. Despite the prediction model failed, this data-driven analysis first validated these associations and identified glucose metabolism markers and dynamic steroid testing as priority targets for larger subsequent studies.

### 4.2 Targeted therapy

In the fields of molecular targeted therapies, ML has provided clinicians with novel insights.​​ Yu et al. integrated 3 ML algorithms—least absolute shrinkage and selection operator (LASSO), support vector machine recursive feature elimination (SVM-RFE), and RF—to screen out five critical APA-associated genes (*SST*, *RAB3C*, *PPY*, *CYP3A4*, and *CDH10*). Using these biomarkers, they constructed an artificial neural network (Yu et al., 2023). The model demonstrated perfect diagnostic performance (AUC = 1.0) in the training sets (GSE156931 and GSE60042) and achieved an AUC of 0.755 in independent validation cohorts (GSE64957 and GSE8514), significantly improving early APA detection. Further drug target analysis via the Enrich platform revealed potential therapeutic agents, including melatonin, phenobarbital, and trichostatin A, with molecular docking confirming their high binding affinity to target genes. Their study not only provides a novel biomarker panel for APA diagnosis but also highlights multi-target therapeutic potential through drug repositioning, offering a foundation for personalized treatment and outcome improvement.

Sun et al. pinpointed *ATP2A3* as a key APA-associated gene through LASSO and RF screening of public RNA-seq datasets (GSE8514, GSE60042, GSE156931), achieving an AUC of 0.879​​ in an independent external data set (Sun et al., 2025). Through spatial transcriptomics, *in vitro* experiments, and immunohistochemistry, they demonstrated that *ATP2A3* upregulates both *CYP11B2* gene expression and CYP11B2 enzyme activity. *CYP11B2* gene is the key gene encoding aldosterone synthase and CYP11B2 enzyme is the key enzyme catalyzing aldosterone synthesis. Their mechanistic studies further revealed that *ATP2A3* mediates these effects through its regulation of intracellular Ca^2+^ homeostasis. They also found that calcium-signaling inhibitors (nifedipine, MCU-i4, W-7 hydrochloride) can significantly​ enhance the suppression of CYP11B2 upon *ATP2A3* silencing. Overall, this study not only elucidates the pathophysiological significance of *ATP2A3* as a novel molecular marker for APA, but also provides a new therapeutic target for treating APA by targeting calcium signaling pathways. Nevertheless, several limitations should be noted: the small spatial transcriptomics cohort (n = 6) may underpower mutation-specific analyses, the precise mechanisms of *ATP2A3*-mediated *CYP11B2* regulation remain unclear, and clinical follow-up data are currently lacking.

## 5 Summary

Currently, the applications of AI demonstrate significant advantages and considerable potential in the diagnosis and management of PA. AI models accurately detect complex PA characteristics by integrating imaging, biochemical markers, and steroid metabolomics, resulting in enhanced diagnostic sensitivity and specificity. ML-based predictive algorithms precisely distinguish unilateral from bilateral subtypes while reducing reliance on traditional invasive examinations, and can guide personalized treatment decisions.

However, current research mainly focuses on PA diagnosis, treatment strategies and prognostic outcomes remain limited. At the same time, the application of AI models faces several challenges: (1) Model training requires high quality and standardized clinical data, as data heterogeneity and small sample sizes may limit generalizability; (2) Current AI models lack transparent decision-making processes and typically require external web-based platforms for clinical deployment; (3) Existing studies primarily rely on retrospective single-center datasets, necessitating prospective validation and multicenter studies to confirm broader applicability; (4) AI-assisted diagnosis requires clear accountability frameworks, particularly when erroneous recommendations cause adverse outcomes, particularly when erroneous treatment recommendations lead to adverse clinical outcomes; (5) AI development necessitates large-scale clinical data acquisition, posing substantial privacy and ethical compliance challenges.

Future research should prioritize algorithmic innovation and cross-disciplinary cooperation to standardize and facilitate the clinical translation of AI in PA management. Specifically, the following key directions should be prioritized: (1) optimizing model algorithm for primary care settings to enhance screening accessibility and initial diagnostic accuracy; (2) integrating advanced multimodal radiomics and genomics technologies in tertiary centers to improve the precision of diagnosis and classification; (3) establishing cross-tier data systems to improve treatment algorithms and prognosis models, thereby building a comprehensive AI-assisted diagnostic framework.

## Glossary

AI: artificial intelligence
PA: Primary aldosteronism
APA: aldosterone-producing adenoma
IHA: idiopathic hyperaldosteronism
ML: machine learning
DL: deep learning
NLP: natural language processing
PAC: plasma aldosterone concentration
ARR: aldosterone-to-renin ratio
AUC: area under the receiver operating characteristic curve
RFr: random forest regression
BMI: body mass index
UPA: unilateral PA
ACEIs: angiotensin-converting enzyme inhibitors
ARBs: angiotensin receptor blockers
MRAs: mineralocorticoid receptor antagonists
RF: random forest
SVM: support vector machine
WSRF: weighted-subspace random forest
XGBT: extreme gradient boosted tree
GMLVQ: generalized matrix relevance learning vector quantization
SIT: saline infusion test
CCT: captopril challenge test
PACT: Primary Aldosteronism Confirmatory Testing
LR: logistic regression
LDA: linear discriminant analysis
LightGBM: light gradient boosting machine
EHR: electronic health record
CT: computed tomography
MRMR: maximum-relevance minimum-redundancy
FNN: feedforward neural network
NNet: neural network
KNN: K-nearest Neighbor
AVS: Adrenal venous sampling
BPA: bilateral PA
GBDT: gradient boosting decision trees
PASO: Primary Aldosteronism Surgical Outcome
DDD: defined daily dose
SHAP: Shapley additive explanations
XGBoost: extreme gradient boosting
OGTT: oral glucose tolerance test
LASSO: least absolute shrinkage and selection operator
SVM-RFE: support vector machine recursive feature elimination
